# Application of a Low-Cost Electronic Nose for Differentiation between Pathogenic Oomycetes *Pythium intermedium* and *Phytophthora plurivora*

**DOI:** 10.3390/s21041326

**Published:** 2021-02-13

**Authors:** Piotr Borowik, Leszek Adamowicz, Rafał Tarakowski, Przemysław Wacławik, Tomasz Oszako, Sławomir Ślusarski, Miłosz Tkaczyk

**Affiliations:** 1 Faculty of Physics, Warsaw University of Technology, ul. Koszykowa 75, 00-662 Warszawa, Poland; pborow@poczta.onet.pl (P.B.); Rafal.Tarakowski@pw.edu.pl (R.T.); przemyslaw.waclawik@pw.edu.pl (P.W.); 2 Forest Protection Department, Forest Research Institute, ul. Braci Leśnej 3, 05-090 Sękocin Stary, Poland; T.Oszako@ibles.waw.pl (T.O.); S.Slusarski@ibles.waw.pl (S.Ś.); m.tkaczyk@ibles.waw.pl (M.T.)

**Keywords:** *Phytophthora*, *Pythium*, e-nose, odor classification, artificial olfaction, electronic aroma detection, volatile organic compounds, fungal and oomycetes volatiles, forest ecosystems, biosecurity

## Abstract

Compared with traditional gas chromatography–mass spectrometry techniques, electronic noses are non-invasive and can be a rapid, cost-effective option for several applications. This paper presents comparative studies of differentiation between odors emitted by two forest pathogens: *Pythium* and *Phytophthora*, measured by a low-cost electronic nose. The electronic nose applies six non-specific Figaro Inc. metal oxide sensors. Various features describing shapes of the measurement curves of sensors’ response to the odors’ exposure were extracted and used for building the classification models. As a machine learning algorithm for classification, we use the Support Vector Machine (SVM) method and various measures to assess classification models’ performance. Differentiation between *Phytophthora* and *Pythium* species has an important practical aspect allowing forest practitioners to take appropriate plant protection. We demonstrate the possibility to recognize and differentiate between the two mentioned species with acceptable accuracy by our low-cost electronic nose.

## 1. Introduction

Many analytical techniques can assess the detection and analysis of odors. Classical chemical analysis methods, which are considered gold standards in analyzing gas samples, often are applied successfully in laboratories. However, due to the high labor and costs related to hiring specialized personnel (e.g., gas chromatography, mass spectrometry), they are not widely used in forestry or horticulture applications. For some such methods, the equipment is not portable (like nuclear magnetic resonance or spectrophotometry), and analysis is time-consuming. There is a need for innovative low-cost instruments allowing fast detection of organisms based on sensors detecting volatile organic compounds (VOCs). They should be suitable for on-site monitoring (meaning that the required time of the measurements is short), and their production costs considerably low. Advances in technology development caused a rapid expansion of artificial intelligent devices, so-called electronic noses (e-noses), as fast and non-invasive diagnostic instruments. Since introducing the e-nose concept [1,2,3], various sensing methods, like optical [4], gravimetric [5], and electrochemical [6], have been developed.

Reviews of the advances in various materials, also useful for e-nose technology and its application in the medical care, food industry, environmental monitoring, and protection, together with the introduction to the algorithms for recognition and classification of the odors, can be found in the articles [7,8]. Achievements and critical evaluation in e-nose systems are reviewed in the paper [9]. The researchers expect dynamic improvement to the electronic noses, which will contribute to odor analysis development.

Advances in e-nose technologies within the plant sciences, including improvements in gas-sensor designs, innovations in data analysis and pattern-recognition algorithms, and material science progress, have led to significant agricultural and forestry benefits. Forestry applications include wood and paper processing, forest management, forest health protection, and waste management. The increase of international trade of plant material (e.g., seeds, potted plants) poses new threats of unintentional introduction of insect pests and fungi into new environments where they can be established and cause dramatic damage to local ecosystems. Problems with forest tree species’ health start at a very early stage of their production in nurseries where seeds are germinated in the soil. Soil-borne fungi genera *Fusarium*, *Rhizoctonia* and *Cylindrocarpon* as well as oomycetes *Phytophthora* and *Pythium* cause damping-off disease reducing significantly the amount of seedlings and causing economic losses. In other cases surviving plants can still carry pathogenic inoculum like chlamydospores among root systems in the soil but not showing the external disease symptoms (i.e., asymptomatic plants). Diagnostic tests performed in many countries showed that seedlings infection in Europe is high and even sometimes reaches 80 percent [10]. Therefore, most fungicides are not intended to limit oomycetes, so their identification is crucial for designing their control adequately due to the emergence of fungicide tolerant isolates of oomycetes in horticultural and agricultural settings [11,12,13]. Moreover, knowing the particular place of their occurrence in a nursery and their potential hosts allow managers via proper crop rotation to avoid any potential infection of plants (e.g., they can grow oak acorns where *P. alni* was found and vice versa alder seeds where *P. quercina* was recorded). However, pathogenic oomycetes are often transferred to other environments (e.g., riparian) together with asymptomatic seedlings being planted along rivers. Plant health inspectors and scientists armed with such new efficient tools like e-noses will be more efficient in taking early action.

A comprehensive comparison and summary of possible applications, challenges, and potential improvements of e-noses focusing on bacterial, fungal, and viral infections, along with their advantages and limitations, are described in review papers [14,15,16,17]. Much of the research to date on fungal aroma compounds [18] has focused on their food and flavor properties. Research on volatile fungal compounds has also taken place to detect plant and animal diseases reflecting their health status. Some other research in similar fields of fungal odors detection should be acknowledged. Recently Guo and coworkers [19] reported studies of *Penicillium expansum* spoilage of apples, Capuano et al. [20] studied *Aspergillus* species discrimination using a gas sensor array, Loulier et al. [21] studied various fungi species using gas chromatography and a differential electronic nose device. Wang et al. [22] studied volatile organic compound emitted by *Phytophthora cactorum* infected strawberries by a newly constructed bioelectronic nose based on single-stranded DNA and a single-walled carbon nanotube. Another fungal infection of strawberries was reported by Greenshields et al. [23]. Detection by the electronic nose of fungal contamination of wood has been reported [24,25]. Other authors reported studies of detection of fungal infection of various grains [26,27,28,29,30,31,32]. Gębicki and Szulczyński [33] presented discrimination of several fungi species based on their odor profile. There are also reports of electronic nose application studies for the detection of fungi in tree roots [34,35]. Sahgal et al. [36] reported discrimination between dermatophyte species and strains. However, due to our best knowledge, the electronic nose’s applications to detect odors of fungi or oomycetes belonging to the forest environment are still in their early stage.

Currently, the simplest method to detect oomycetes in the soil is baiting [37]. It allows these organisms to grow, for example, on oak and/or beech leaves, with the infected leaf pieces, then plated into selective media (e.g., PARP) [38]. Such an approach requires several days or weeks to get pure cultures of pathogenic oomycetes to identify them with classical (microscopic) or molecular (DNA sequencing) methods. Baiting can be complicated when one has to use various host leaves, and isolation temperatures need to vary for different organisms. Metabarcoding indeed collects a higher diversity of oomycetes in soil samples but is also not without its limitations. This paper focuses on the creative possibilities of a new, innovative tool for forest practitioners, allowing them to detect pathogenic oomycetes in forest nurseries, plantations, and high forests.

The practical application of an electronic nose in agriculture and forest industries require the affordable price of the device. Thus relatively simple constructions may have an advantage over much sophisticated but costly devices, providing the accuracy of odor detection and classification is sufficient. In recent years there have been many proposals for the construction of low-cost electronic noses. Several groups proposed devices based on Taguchi type MQ series gas sensors [39,40,41,42,43,44,45]. Oates et al. [46] presented research on a sinusoidally heated e-nose applied to olive oil type classification. Recently, several constructions were reported by Gonzalez Vieyo et al. [47] to assess aroma profiles of beer, by Anyfantis and Bliona [48] to detect human presence, by Wu et al. [49] for cigarette brand identification, and by Szczurek et al. [50] to detect bee colony infestation. Development of a portable electronic nose for detection of pests and plant damage has been reported by Lampson and coworkers [51].

This paper is organized as follows. In the next section we explain the main motivations of the performed studies, in Section 3 we describe materials and methods, including oomycetes cultivation and samples preparation (Section 3.2), construction of our e-nose device (Section 3.1), measurement procedure (Section 3.3) and data processing and building of machine learning models (Section 3.4). Then, in Section 4 the results of our modeling are discussed. In Section 5 we summarize our findings.

## 2. Motivation of the Research

In our experiment, we wanted to face the context of biosecurity, keeping in mind our objective, which is the early detection of emerging diseases caused by oomycetes, especially in the nursery. It has been reported [52] that *Phytophthora* species, due to their emitted odors, can detect trained dogs by sniffing. It would be helpful to use an artificial device for this task. We understand that this goal is ambitious. Therefore the first experiments were designed and performed in vitro in controlled laboratory conditions. This time we managed to show that our designed e-nose can discriminate between two different closely related oomycetes. This issue is not common evidence for the comparisons between *Phytophthora* and *Pythium* specimens because even molecular tests, for example, ELISA, often give false-positive results if microorganisms exist in the same environment, for example, soil. Our challenge is to demonstrate the efficacy of discrimination in a semi-natural or natural system like a forest nursery. To reach this goal, we started with the experiment to discriminate between two oomycetes species, which are difficult to discriminate in practice, for example, microscopic work. In the next step, we will set an experimental design in microcosmos, at least with a range of different species belonging to more than two genera, and using the data sets for solid training. This design would better simulate the reality of a highly biodiverse community of oomycetes in an artificial environment such as a nursery and provide a base for further analyses and experiments in mesocosms conditions (e.g., infected plants in greenhouse experiments). Even then, the whole system’s complexity would be missed (with host plants but not the whole soil-borne microbial community), but at least a baseline of information could be acquired from the mesocosms experiment. It is the first step, which applies a methodology, this time to two well-known model microorganisms (*P. plurivora* and *Py. intermedium*), with a direct connection to the objectives of using the system for early detection in the future as a part of larger biosecurity system.

## 3. Materials and Methods

### 3.1. Electronic Nose Device

**Figure 1:**
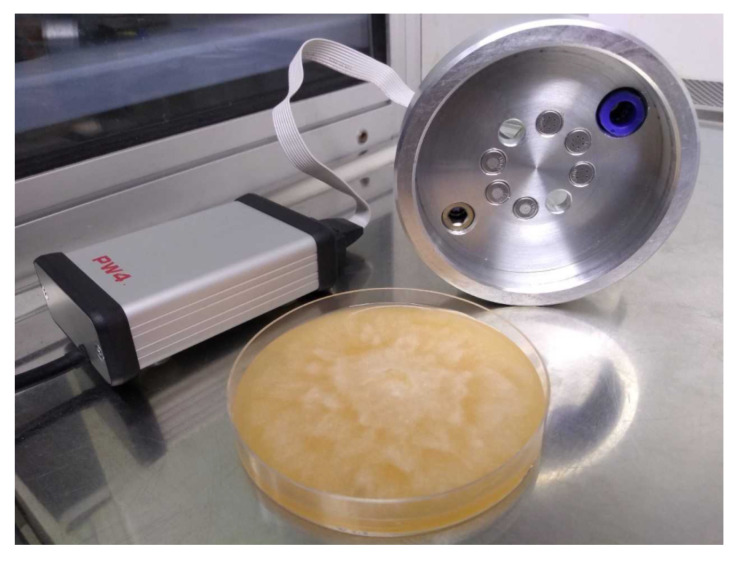
Electronic nose device with a measured sample in a Petri dish.

The main component of an electronic nose device is a set of non-specific gas sensors. In our construction, we used six metal oxide sensors produced by Figaro Inc. (Osaka, Japan). The types of applied sensors are listed in Table 1. Each of these sensors responds to various types of gases with different characteristics. Thus detection of a specific gas can be achieved by comparing responses of multiple sensors or examining the complete response of a given sensor to a change of gas conditions (turning on/off between reference clean air and gas), with the help of machine learning techniques. Sensors are mounted in a metal probe, which can be moved manually and placed close to the measured odor. This probe prevents touching the measured sample and allows to maintain a small volume of air between sensors and the sample. This air volume has 81 mm in diameter and 12 mm in height. The probe’s dimensions fit the Petri dish as it is presented in Figure 1.

We decided to simplify the device as much as possible without using a sophisticated sensor chamber and gas supply system with pumps and valves. One of the main reasons was reducing the cost and verification if such a device could collect useful odor recognition data.

Sensors are connected to an MCP3208 12-bit AD converter. The sensors respond to the gas by changes of conductance/resistance. To measure the sensor’s response, we implemented a voltage divider circuit for each sensor, and we measured the voltage on the serially connected resistor. We chose this measurement method due to its simplicity and because the sensors producer recommends it. However, also other topologies of measurement circuits can be implemented for the MOS-type electronic noses [53]. For each sensor, the voltage probe took about 20 ms and was repeated 50 times and averaged to reduce the noise. This operation was performed in a loop for each sensor. The reading of the averaged voltages from all sensors took about 1.22 s. Output data were stored online in the text file. All control of the device’s operation and collection of data was performed on the laptop. The device’s power supply was also provided by the connection to the laptop computer via USB. Such a design was chosen for reduction of cost of the potential device, without the need to use additional electronic components for functionalities that can be performed by the laptop computer. More details concerning the constructed electronic nose hardware are available in the Supplementary Materials.

### 3.2. Cultivation of Oomycetes and Samples Preparation

The strains of oomycetes were isolated in a forest nursery from European oaks *Quercus robur* (KX242301) and were kept in the stock of the Department of Forest Protection in the Forest Research Institute in Sękocin Stary (Poland). For detailed analysis, *Phytophthora plurivora* was chosen as the most abundant pathogen in forest nurseries, and stands in Poland [54] as well as in other countries, for example, Austria [55]. As a similar species (but not so pathogenic) *Pythium intermedium* (MN255129) was proposed for comparative studies [56]. Both organisms were cultured on classical V8-Agar media (16 g agar, 2 g CaCO_3_, 200 mL vegetable juice, and 900 mL distilled water) in 9 cm Petri dishes. They were kept at room temperature until the mycelium overgrew the whole surface of a dish.

### 3.3. Odor Measurement

The performed experiment of measurements of odor samples consisted of two parts. The first part of the measurements took place between 29 October and 13 November 2020. We used five samples of each studied culture during this part of the measurements, prepared as described in the previous section. These measurements are intended to collect data that will be used for further exploration and building learning classification models for the device used for differentiation between studied oomycetes.

The second part of the experiment was performed between 24 November and 27 November 2020, and for these measurements, we used two samples (two Petri dishes) of each studied culture. The purpose of these measurements was to collect a dataset of independent observations that could be used to evaluate the performance of the created device learning model used to detect the pathogens. For that reason, the cultures were prepared in an independent process with a newly prepared growth medium.

The measurements during a day were performed in series. In one series, we always used two samples of each kind of the measured organisms and two samples of the pure medium as a control variant, meaning six measurements in a sequence. During a series of measurements, the samples’ order was randomized using a random number generator from an Excel spreadsheet. The purpose of randomization was established to avoid possible bias and discover patterns that could occur due to trends in measurement setup or external conditions rather than due to the natural variability between studied samples.

During one day, we usually performed two series of measurements. There were two days when three series of observations were collected, and four days when only one complete series or even less was collected. Such design of the data collection process was intentional. During a day of measurements, we could observe that the response of an electronic nose exposed to a given sample type was similar. More prominent variability could be noticed when the same sample was measured on different days, which could be attributed to slightly changing conditions of measurement or changes in the sample itself due to the culture’s growth. For that reason, we assumed that collecting too much data during one day of measurement could lead to overestimation of the recognition performance, and extending the time of data collection would lead us to more reliable results. In total, in the first part of the experiment, we performed 108 measurements, and in the second part of the experiment, 48 measurements.

**Figure 2:**
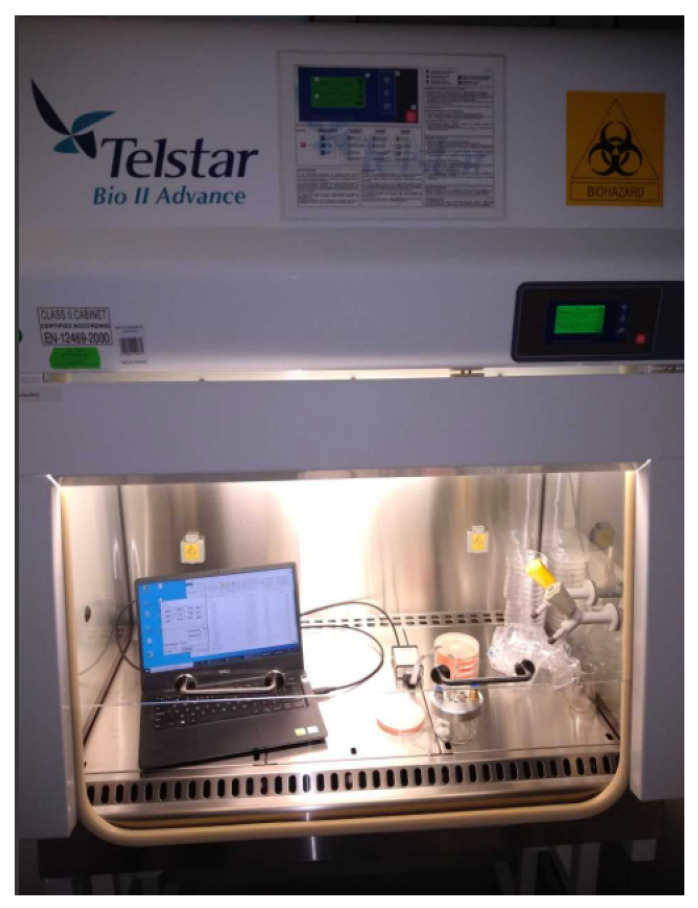
Measuring station with the low-cost electronic nose attached to a laptop inside the laminar flow cabinet.

We conducted all measurements under the laminar flow cabinet (Telstar, Bio II Advance) with the air supply turned on and at the temperature of 21 °C. The measurement setup is presented in Figure 2. At the beginning of the measurement, we exposed the sensors to clean air and continued collecting the baseline responses of sensors for 100 readings of the values, which lasted 122 s. Then, we opened the dish with the pathogen and put the sensors into the dish. The change of lids is concise, which allows for a quick change of the conditions in which the sensors operate from clean air to measured gas. That gas adsorption process in the e-nose sensors array lasted another 100 readings (122 s). After that, we manually moved the sensors array back into clean air and covered the dish again. We collected the desorption phase of sensors response during relaxation in clean air for further 500 readings (610 s). In this measurement procedure, we used air present in the laminar flow cabinet as clear enough for establishing baseline conditions to clean the sensors after exposure to the measured odor. This procedure is not as precise as in a fully automatic device with a gas and clean air supply system of pumps and valves, but it turned out to be sufficient for our purpose. Gonzalez Viejo and coworkers [47] have recently demonstrated a similar construction.

Another point to notice is that samples were kept sealed, which prohibited accidental contact with other organisms and their growth on the Petri dish and the studied culture. To prevent such accidental infection from happening, we decided that after the opening of a Petri dish for measurements, this particular sample was used no longer than three consecutive days.

The electronic nose device was connected to the electric power without interruptions, and when measurements were not performed, sensors were placed in a jar in clean air. Powering the device was necessary to maintain the operation of sensors heaters. Also, as is recommended by the producer, sensors were preheated before the experiment for at least seven days.

### 3.4. Classification Modeling

The differentiation between samples of odor measurements using electronic noses is a classification task using machine learning models [57,58,59]. Such action is performed following a well-established methodology consisting of multiple tasks: (i) transformation of data with the aim of extraction and engineering of modeling features, (ii) selection of the reliable modeling features, (iii) machine learning modeling, (iv) assessment of models performance, (v) selection of the final model (vi) and, visualization of the data and results. It is essential in order to gain additional insights into the performed studies. Finally, (vii) the verification of the modeling results on an independent data set is beneficial.

All the data processing and machine learning tasks presented in this paper were performed using computer codes developed in Python 3.7 language, with the scikit-learn module [60].

#### 3.4.1. Modeling Features

The electronic nose data collected are a series of sensors’ responses expressed as the measured voltage over time, related to Ohm’s law’s electric conductance. As the first step of data processing, the measured data were standardized by the baseline value observed during the first phase of the data collection, when the sensors were exposed to the clean air, treated as the reference ($U/U_{0})$. As the baseline, we used the average of the first 100 observations of the voltage collected by each sensor. This procedure allows reducing signal dependence on the sensor drift during the whole time of the experiment.

In the next step of data processing, various features describing shapes of the measurement curves were extracted from the data. Such actions include characteristics itemized below.

- Basic statistics calculated from the whole response curve, for example, maximum, sum, standard deviation.
- Time needed to reach the indicated percent change of the sensor response.
- Extreme values of exponential moving average filter, characteristic times needed to reach these extremes, and response values at the moment when the extremes are reached.
- Basic statistics calculated for the exponential moving average filter.
- Parameters of sensor response curve fitting by the 3rd order polynomial.

What is more, these features are calculated for the whole sensor response curve and the adsorption and desorption phases separately. The full list of the features that we have used for training classification models is presented in Appendix A in the other report [61].

#### 3.4.2. Machine Learning Models Building and Testing

As a machine learning algorithm for classification, we used the Support Vector Machine (SVM) method with the Radial Basis Function (RBF) kernel, which is often applied to electronic nose data. We had tested other methods such as logistic regression or k-nearest neighbors, but we had not observed improvement of the results for the considered dataset.

We decided to use only such classical modeling techniques and disregard more complex algorithms such as multi-layer neural networks since in the dataset that we prepared, the number of observations is quite limited. Collection of the additional data is lengthy and costly in terms of the working hours. Even though more flexible modeling techniques can provide more expressive classification models, the number of fitted parameters would be much higher than in the method we applied. For example, for the case of neural networks, some authors [62] (p. 25) indicate that the total number of training data points should be at least 2 to 3 times larger than the number of the network parameters. However, the precise number of training data instances depends on the specific model at hand. Hence, in principle, simpler models should be less prone to over-fitting.

An essential part of the modeling process is variable selection, for which we implemented a recursive forward selection procedure. At the first step, we built models just on a single feature, and the best of them were selected according to the accuracy measure. In the following step, we compared models built on two features, one previously selected and the second in a loop of all remaining. This procedure was repeated recursively.

The data set of measurements that we collected is quite limited. Hence splitting it to separate training and testing partitions would reduce its size too much. To deal with this issue, we decided to apply the commonly used cross-validation procedure (CV).

The variable selection was performed in a double loop of CV, in which data were split into testing and training partitions. Firstly, in the external CV loop, the available data set was split into a testing partition consisting of data measured during one particular day. Measurements from the other days consisted of the second data set used for models training for variables selection. This data set was again split into two sets in the internal CV loop, using the same grouping-by-day data measurement approach. The model was trained on the training data set, and their performance assessed on the validation data set, consisting of data from one day of measurements. The classification accuracy averaged over partitions from the internal loop was used to select the best set of modeling features. The results reported in this paper are averages over partitions of the external CV loop. That guarantees that the data used to evaluate the model performance were separated from the model training and variables selection data.

There are a few other details of this procedure that should be noted. We decided to use CV in groups to avoid correlations between data records, which could lead to overestimating the classification performance. As we visualized the measurement curves, there were similarities between measurements performed during one day, even when we considered different samples of the same type. More variation was observed when looking at measurements performed on different days of the same sample. This variation could be attributed to the aging of samples or unavoidable differences in the environment. For that reason, we chose to group data by day of measurement when partition on training and testing data sets were performed. The number of measurements per day was not the same, and to account for this variability, we used a weighted average. Another important observation that should be recalled is that there could be a different set of modeling features in the external CV loop for each day’s testing partition.

#### 3.4.3. Measures of Classification Performance

Various quantities can be used to assess the performance of classification models. In the present work the following measures are used.

- The first that we used is accuracy, which is defined as the proportion of the correctly classified observations to the total number. However, also, other measures can be used and can give insightful information about the studied case.
- The second measure of the models’ performance, used in our work, is *recall*, which should be calculated for each of the considered categories. It is defined as the ratio between the number of correctly classified observations of a given category and the total number of observations in this category. This measure is focused on the possibility of detection of observations belonging to this category and is not penalized by cases when observations from other categories are incorrectly classified as that one.
- The third measure of the models’ performance used in our work is *precision*, which is also calculated separately for each classification category as a ratio of the number of correctly classified observations and the number of observations classified to this category. That means that this measure is focused on the confidence that the classified observation truly belongs to this category.

## 4. Results and Discussion

### 4.1. Sensors Response Characteristics

The results obtained by the electronic nose measurements are response curves of the sensors array when they are exposed to the studied odor and when they recover to the baseline after exposure to the clear air.

**Figure 3:**
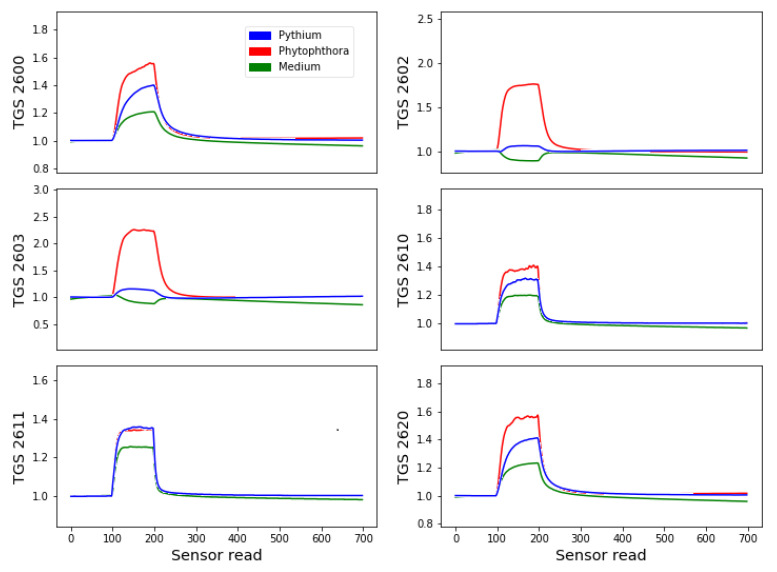
Examples of sensor response versus time of measurement (number of reading during measurement) for each sensor in the sensor array. Separate curves for three studied sample types are presented. *Y*-axis represents the sensors’ responses normalized by the baseline value, calculated as $G/G_{0}$, where *G* is the measured conductance, and $G_{0}$ is the average of the conductance measured in the clean air. On the *X*-axis, the numbers of sensor reads are shown, which happens every 1.22 s.

In Figure 3, we present an example of measurements of various studied sample types during one day. As we noticed in Section 3.4.1 for the analysis, we do not use the original value of sensor response but its proportion related to the baseline, which is the response of the sensor exposed to the clean air in the first stage of measurement. As one can notice in this figure, during the measurement phase, there is an apparent flat response curve during the baseline collection, then noticeable response change when sensors array are placed close to the studied sample and again recovery to the baseline when the sample source was removed.

The choice of the examples presented in Figure 3 is arbitrary. We intended to present the character of the shape of response curves. We noticed that one species’ response curves were similar and distinct from other species during one day of measurement, even compared by the naked eye. More variability was observed between various days of measurements, which we could attribute to slightly different measurement conditions, but also the aging of measured samples as one sample (one Petri dish with a culture) was measured during a few days. However, this variability should be accepted, as in real conditions, it is inevitable. More examples of the sensor’s response during several days, demonstrating the sensors drift, are provided in the Supplementary Materials.

As one can notice, during the desorption phase of the measurement, the time when the sensor response reaches the saturation magnitude can be different for different sensors. For example, for the sensor TGS 2611, it is about 15 sensor reads (18 s), but for sensor TGS 2600 during 122 s (100 sensor reads), when the sensors were exposed to the measured gas, there is still an observed rise of the response, which means that sensors may not reach the saturation. We mention here the TGS 2600. However, for other sensors, it also may happen. We also observed that the time of sensors response to the measured odor and sensors recovery time are similar. However, due to the long tail and slight asymptotic decrease, we decided to wait longer to avoid “memory effects” when previous samples’ measurements strongly influence the measured values. We also need to admit that we noticed a few cases when the assumed recovery time in the clean air of 610 s was insufficient for some sensors. We have not identified the reasons for such behavior. We kept these outliers for further analysis, including the classification modeling, even if it could lead us to report less optimistic results.

We examined if this influences our studies’ classification results for further analysis, including classification tasks. We calculated a series of modeling features extracted from the response curves, in which data only from a part of the response were used for various times of data collection. That was equivalent to using only information from the desorption phase. As we verified, there was no significant improvement in the classification results, described in the following sections, when the sensor’s exposure to the studied odor was at least 40 sensors reads (49 s). We note that such an analysis could be done only after collecting the whole dataset and comparing the classification performance results.

Recently, Rodriguez Gamboa and coworkers [63] reviewed studies on the possibility of using a much shorter time of data collection; however, as we verified, such an approach is not relevant for our e-nose device and/or types of odor.

We removed one day of collected measurements as they were affected by the external odor of an open bottle of disinfection alcohol, which was visible in the charts.

### 4.2. Principal Component Analysis

**Figure 4:**
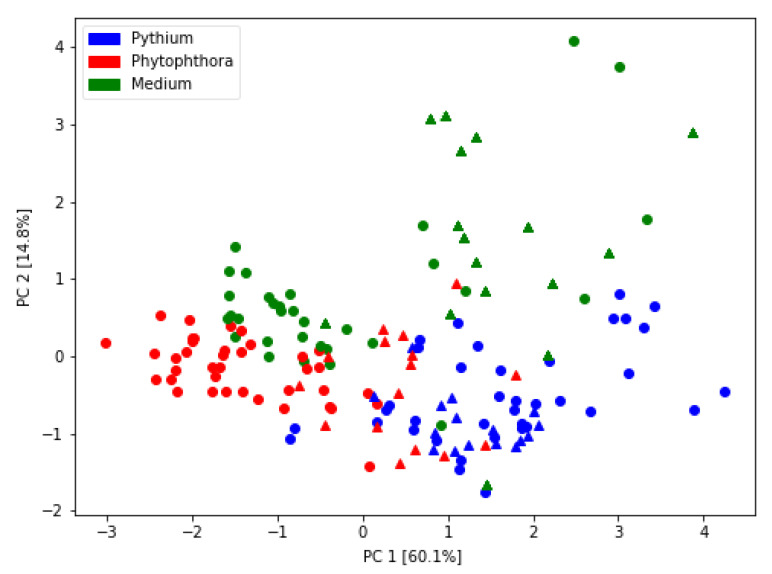
Visualization of measured samples distribution using Principal Component Analysis transformation of five features extracted from the sensor response curves. The principal components’ percentage of variability is indicated in the axes captions. Types of measured samples are plotted using various colors. As described in the text, data collected in two parts of the experiment are distinguished by different symbols: circles—the first part, triangles—the second part.

In Figure 4, we present the PCA transformation of the studied dataset when as input, we used five features selected by the classification algorithm. There are a few interesting patterns that we observe in this figure.

First of all, there is a distinct separation of the points belonging to two studies species of oomycetes (*Phytophthora* and *Pythium*). In this figure, we differentiate by different symbols, two parts of the experiment performed in two distinct time periods, using independently prepared culture samples. We do not find any apparent patterns of separation of data from these two parts of the experiment for the grown cultures. This observation is an encouraging sign that the pattern of separation of *Phytophthora* and *Pythium* is likely due to these species properties.

There is a critical observation that can be noticed in this figure regarding the distribution of measurements of the growth medium samples. These points are still separated from the studied oomycetes. However, we notice the separation of the data from the first and second part of the experiment. We suggest that these two media were slightly different due to variations in the ingredients, such as V8 juice. In our opinion, this is encouraging for the global goal of our research as it means that oomycete cultures grown on different media are similar in the PCA picture and that the found patterns of the differentiation between *Phytophthora* and *Pythium* are caused by the odors emitted by these species and registered by the electronic nose. That increases the likelihood of the generalization that the differentiation between these species using an electronic nose and trained machine learning model will hold for newly collected data.

### 4.3. Results of Machine Learning Classification Models

#### 4.3.1. Multiclass Classification Model

We used data collected from all six sensors available in the electronic nose in the first model that we trained. The main measures of the classification performance are presented in Table 2. What can be noticed is that the accuracy and other measures calculated by the data collected in the first phase of the experiment, by the cross-validation method, exhibit the high performance of classification of the studied oomycetes, for all considered measures at least 90%.

It is not surprising that the model performance assessed using the independent testing dataset is lower than the one obtained by the cross-validation method. However, as one could notice, the results of the recall of *Phytophthora* are meager. That may be explained by the fact that the built model is optimized to reach maximum accuracy based on the training dataset. To achieve that, it considers the patterns in the whole dataset, with all included categories. As we discussed in Section 4.2 the patterns of data collected for growth medium are remarkably different, leading to the classification boundary between medium and other categories, which is not sufficiently generalizing for new datasets.

#### 4.3.2. Classification Model for Differentiation between Two Studied Oomycetes

The results presented in the previous section may seem discouraging, as the recall of the *Phytophthora* measure is the most important when one thinks about potential applications in forestry to detect the presence of this species in the environment. However, often we are interested in a different type of classification. We are looking preferably for differentiation between the two most popularly encountered species, as in the present studies of *Pythium* and *Phytophthora*. For such a case, we need to build a machine learning model with a binary target.

In Table 3, we present the results of such models built on the same data sources as described when the observations belonging to the growth medium category have not been included. In this case, we achieve an almost perfect classification of data when the assessment measures were estimated using the cross-validation method on the dataset collected in the first part of the experiment. The differentiation between the studied species on the independent dataset has lower power since the detection of *Phytophthora* is slightly better than that of *Pythium*. However, such a level of accuracy is often acceptable for preliminary, low-cost tests.

#### 4.3.3. Differentiation between Studied Samples Using Data from Reduced Sensors Array

The further study that we performed using collected data is a verification of the possibility of further reducing the price of the low-cost electronic nose that we construct. As we demonstrated using different datasets [61], it may be feasible to reduce the sensor array and retain only a small portion of sensors, or even one sensor. Despite such reduction, the classification models’ performance, built on features extracted from the dynamic sensors response curves, allows achieving a more similar classification performance than the one obtained using the data collected by all sensors.

We verified the models’ performance for each sensor and noticed that the TGS 2602 sensor data exhibited the best classification performance. In Table 4, we present results of such models, built using only data collected by this sensor. As one can notice, these results are very similar to the ones presented in Table 2 and Table 3, where data from all sensors were used.

The TGS 2602 sensor is designed to detect the odor and air contaminants [64]. It has a high sensitivity to low concentrations of odorous gases such as ammonia and H_2_S generated from waste materials in office and home environments. Bacteria and fungi often cause food waste, and for that reason, the characteristic of this sensor’s response is designed to detect metabolites emitted by such kinds of species [18]. In our research, we applied data from that sensor to fungi-like organisms that do not cause food spoilage but usually survive in plant remains for many years. We demonstrated that using machine learning models based on this sensor data can distinguish between two species of oomycetes species encountered in the forest environment.

## 5. Summary

An electronic nose is a very much wanted tool by the managers of forests and ornamental nurseries. Our investigations could be considered as the first steps to reach this goal. We believe that a quick detection of pathogens during the production of regeneration material (seedlings of different forest species) is the best strategy to undertake the appropriate protection measures. Early warning is a key issue in designing the appropriate preventive plan, which is in line with the EU Directive on Integrated Plant Disease Management. The knowledge about pathogens present in a particular nursery plot allows managers to avoid potential infection, for example, by using pathogen-resistant plant species in succession. At first, we concentrated on experiments carried out in laboratory control conditions before tests on inoculated with pathogens potted plants growing in a greenhouse. We wanted to discriminate between less and more pathogenic organisms using as model *P. intermedium* and *P. plurivora*, respectively. Those oomycetes are common soil-borne organisms, especially the latter one is considered alien, invasive species responsible for the emerging disease of many forest tree species. In this context, discrimination by odor will offer managers the unique opportunity of fast selection still in the nursery. When infected (often asymptomatic) renewal material is planted in forest plantations, eradicating alien, invasive species is much more costly and even sometimes impossible. Future sustainability and biodiversity of European forests depend on the quality of nursery material.

In the present paper, we report the results of our first attempts to build a low-cost electronic nose device capable of differentiating between two common forest plants, pathogenic oomycetes *Py. intermedium* and *P. plurivora*. The results are encouraging since it was possible to collect data and build the classification models. In the reported experiment, the electronic nose could differentiate odors of pure cultures cultivated in vitro. Therefore, there is a need to isolate these organisms from the soil, for example, with cheap and effective indicator (sensitive) plants or baiting techniques, which is routinely done in any nursery. Typically, from the grown pure cultures of pathogens, DNA is extracted to identify the species. The electronic nose can shorten this procedure when discriminating species of oomycetes still in the nursery level [65]. It is also essential because most fungicides designed for fungi are not efficient enough to control oomycetes. Some plants do not show any symptoms in the nursery but are not fully recovered (the disease is only suppressed). Under favorable conditions, the spores germinate, and the disease process begins.

Differentiation between *Phytophthora* and *Pythium* species has an important practical aspect allowing forest practitioners to take an appropriate plant protection strategy. Usually, species belonging to the *Pythium* genus are less pathogenic than those belonging to the *Phytophthora* genus. Therefore, knowing what species is in a particular plot in the nursery is crucial for deciding to undertake (or not) and what kind of action. The discrimination between weak and strong pathogens in practice is whether managers use pesticides or not. *Pythium* species are so often abundant in the soil that possibly no action is required while *Phytophthora* is always considered to cause managers’ problems. Those species usually damage fine roots (less than 2 mm of diameter) and the base of stems (like *P. plurivora*) [66], but also they can attack the upper part of plants (*P. infestans* or *P. ramorum*) [67,68]. In this case, action is always required, and the earlier, the better, so early detection systems like e-nose cannot be overestimated.

## Figures and Tables

**Table 1:** List of sensors used in the e-nose device and most sensitive molecules.

Type of Sensor	Main Sensitivity for Gases
TGS 2600	air contaminants
TGS 2602	VOCs, ammonia and H_2_S
TGS 2603	amine and sulfur series odor (trimethylamine, methyl mercaptan, etc.)
TGS 2610	LP gas
TGS 2611	methane
TGS 2620	Organic solvent vapors

**Table 2:** Results of multiclass target classification. Two methods calculate various machine learning models’ performance measures: cross-validation performed on the data collected in the first part of the experiment and tested on the separate dataset of observations collected in the second part of the experiment.

	CV on First Part Data	Test on Second Part Data
Accuracy	90%	71%
Recall of *Pythium*	94%	82%
Recall of *Phytophthora*	95%	38%
Precision of *Pythium*	90%	83%
Precision of *Phytophthora*	92%	76%

**Table 3:** Results of binary target classification. Two methods calculate various machine learning models’ performance measures: cross-validation performed on the data collected in the first part of the experiment and tested on a separate dataset of observations collected in the second part of the experiment.

	CV on First Part Data	Test on Second Part Data
Accuracy	97%	78%
Recall of *Pythium*	94%	67%
Recall of *Phytophthora*	100%	88%
Precision of *Pythium*	99%	74%
Precision of *Phytophthora*	95%	85%

**Table 4:** Results of models trained on data from only one sensor (TGS 6302). Two methods to calculate various machine learning models’ performance measures were used: cross-validation performed on the data collected in the first part of the experiment and tested on a separate dataset of observations collected in the second part of the experiment.

	Multiclass Target Classification	
	**CV on First Part Data**	**Test on Second Part Data**
Accuracy	90%	60%
Recall of *Pythium*	98%	80%
Recall of *Phytophthora*	97%	35%
Precision of *Pythium*	82%	65%
Precision of *Phytophthora*	95%	71%
	**Binary Target Classification**	
	**CV on First Part Data**	**Test on Second Part Data**
Accuracy	100%	75%
Recall of *Pythium*	100%	81%
Recall of *Phytophthora*	100%	69%
Precision of *Pythium*	100%	72%
Precision of *Phytophthora*	100%	79%

## Data Availability

The data presented in this study are available from the corresponding author.

## Supplementary Materials

The following are available online at https://www.mdpi.com/1424-8220/21/4/1326/s1, Figure S1: An electric circuit of signal measurements of the TGS sensor; Figure S2: The inside view of the device; Figure S3: Sensor responses for measurements performed on 2020-11-04. Units of axes and meaning of curves as described in the text; Figure S4: Sensor responses for measurements performed on 2020-11-05; Figure S5: Sensor responses for measurements performed on 2020-11-09; Figure S6: Sensor responses for measurements performed on 2020-11-10; Figure S7: Sensor responses for measurements performed on 2020-11-12; Figure S8: Response of sensors exposed to the clear air during the baseline measurement. Vertical lines separate different days of the experiment; Figure S9: Several examples of sensors’ responses to growth media odors for measurements on various samples during several days; Figure S10: Several examples of of sensors’ responses to Phytophthora odors for measurements on various samples during several days; Figure S11: Several examples of of sensors’ responses to Pythium odors for measurements on various samples during several days.
