# Supplementary material for: Application of a Low-Cost Electronic Nose for Differentiation between Pathogenic Oomycetes Pythium intermedium and Phytophthora plurivora
Source: Sensors (Basel). 2021 Feb 13;21(4):1326. doi: 10.3390/s21041326 (PMC7918289; doi:10.3390/s21041326)
Supplement: Supplementary file 1 [file sensors-21-01326-s001.pdf]

Supplementary Materials: Application of a Low-Cost Electronic Nose for Differentiation Between Pathogenic Oomycetes *Pythium Intermedium* and *Phytophthora Plurivora*

Piotr Borowik [1], Leszek Adamowicz [1,*], Rafał Tarakowski [1], Przemysław Wacławik[1], Tomasz Oszako[2], Sławomir Ślusarski[2], Miłosz Tkaczyk[2]

1. Design of the electronic nose device

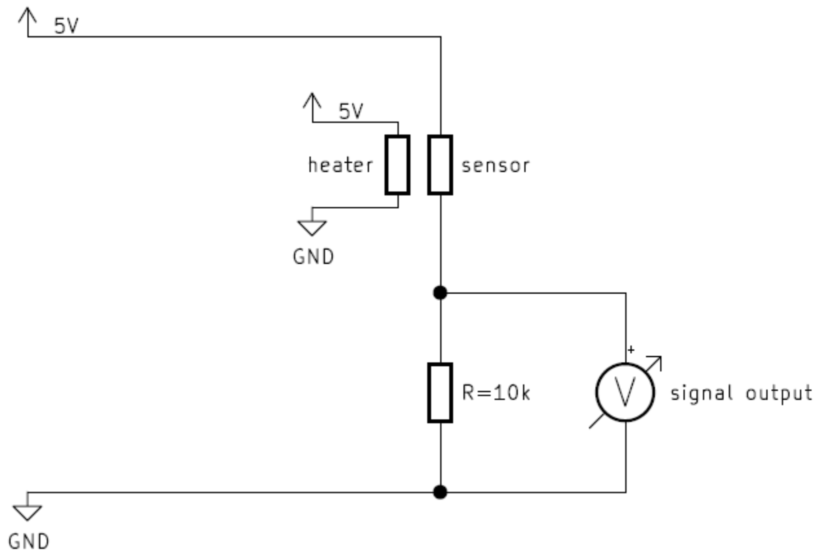

Figure S1. An electric circuit of signal measurements of the TGS sensor.

The measuring part of the device is a TGS type sensor connect in series with 10k Ω resistor and 5 V DC source S1. A heater is an integral part of the sensor but has a separate voltage source. The signal is measure by the MCP3208 AD converter, which uses six input channels. Each channel is connected to a different sensor. A signal is processed by the Atmel ATTiny 2313-20PU microchip and send to the computer via USB cable. The inside of the device is shown in figure S2. The computer is also a power source of the electronic nose.

As in every low-coast device, the most expensive device parts are sensors, which cost no more than $20 each. Compared to the AD converter coasts about $2.50, microchip about $1.50, each of other parts costs less than a single US dollar.

2. Sensor responses

The other supplementary results we want to present are some of the control charts used to examine the measurement data. Those are sensor response curves demonstrating the variability of these measurement results. That may be caused by sensor drift, possible variability of environmental conditions, variability and variability of measured samples, and variability of odors intensity.

In Figures S3–S7 we present the examples of sensors responses measured during several days of the experiment. The first set of subfigures present original reads from the electronic nose's set of sensors. The sensor type is mentioned as the y-axis label. The units of the y-axis we treat as arbitrary

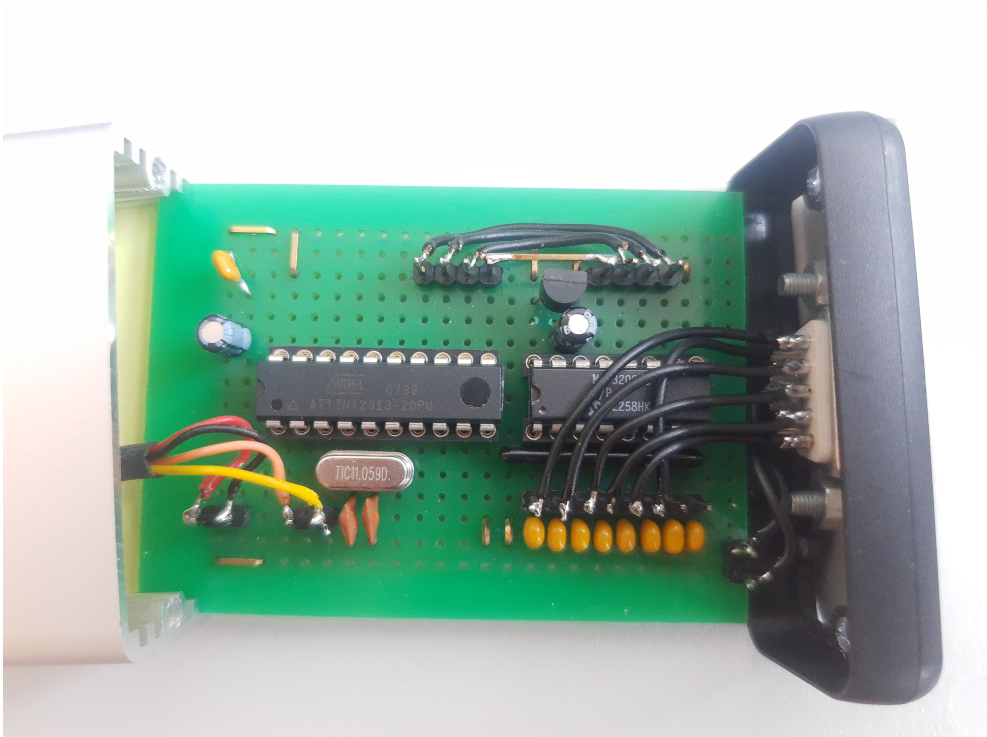

Figure S2. The inside view of the device.

units. However, they are [mV] of voltage measured on 10 kΩ resistor. This resistor is connected in serial connection to the sensor, and this is plugged into the 5V source. We do not calculate the sensor's real resistance or conductance as it is not necessary for our analysis. All data used in the analysis are relative to the baseline value - the value measured at the beginning of each measurement when the sensors are placed in the clean air conditions (U/U_0). Such scaled response curves are presented in the second set of subfigures. In all subfigures on the x-axis, we display the performed measurement hour. We distinguish the sample type - growth medium or genre by different colors. As described in the main text, individual samples measurement was randomized.

As one can notice there can be observed baseline drift even in the timescale of a few hours during one day of measurement. This drift is noticeably visible in Figure S4 for measurements performed on 2020-11-05 by the sensor TGS 2603. One can also notice the baseline drift for other days or other sensors.

In Figure S8 we present trends of sensors' response to the clean air during the whole time of the experiment. We draw only data collected during the baseline measurement, so only first 100 sensor reads before placing the sensor array close to the measured sample. By vertical lines, we separate measurements performed on different days. In this figure, due to the chart's place limitation, we omit the x-axis caption as we think it is not necessary in this case. We intend to present the overall trends and orders of magnitudes of the baseline magnitude variability.

Besides the drift of the baseline, we can also observe substantial variability of the sensor's response to the measured odors. In Figures S9,S10,S11 we present examples of sensors' responses to odors for measurements on various samples during several days.

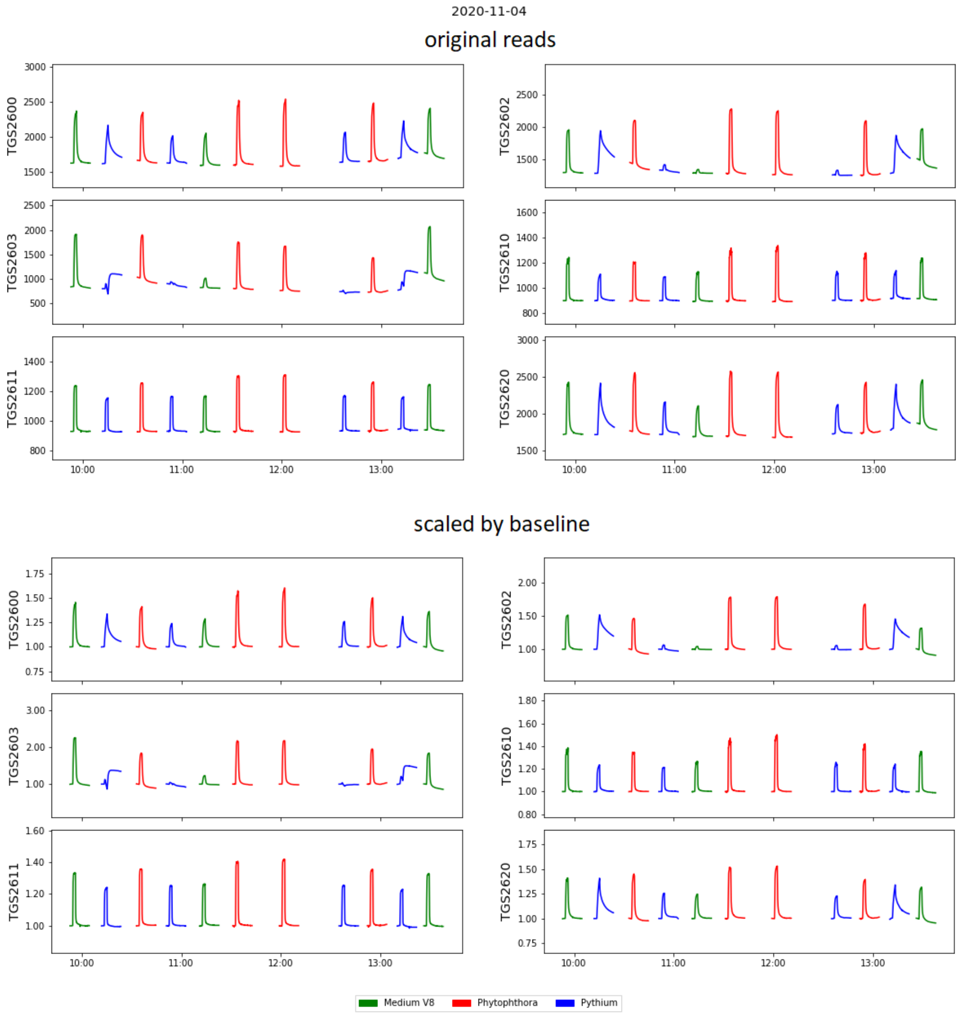

Figure S3. Sensor responses for measurements performed on 2020-11-04. Units of axes and meaning of curves as described in the text.

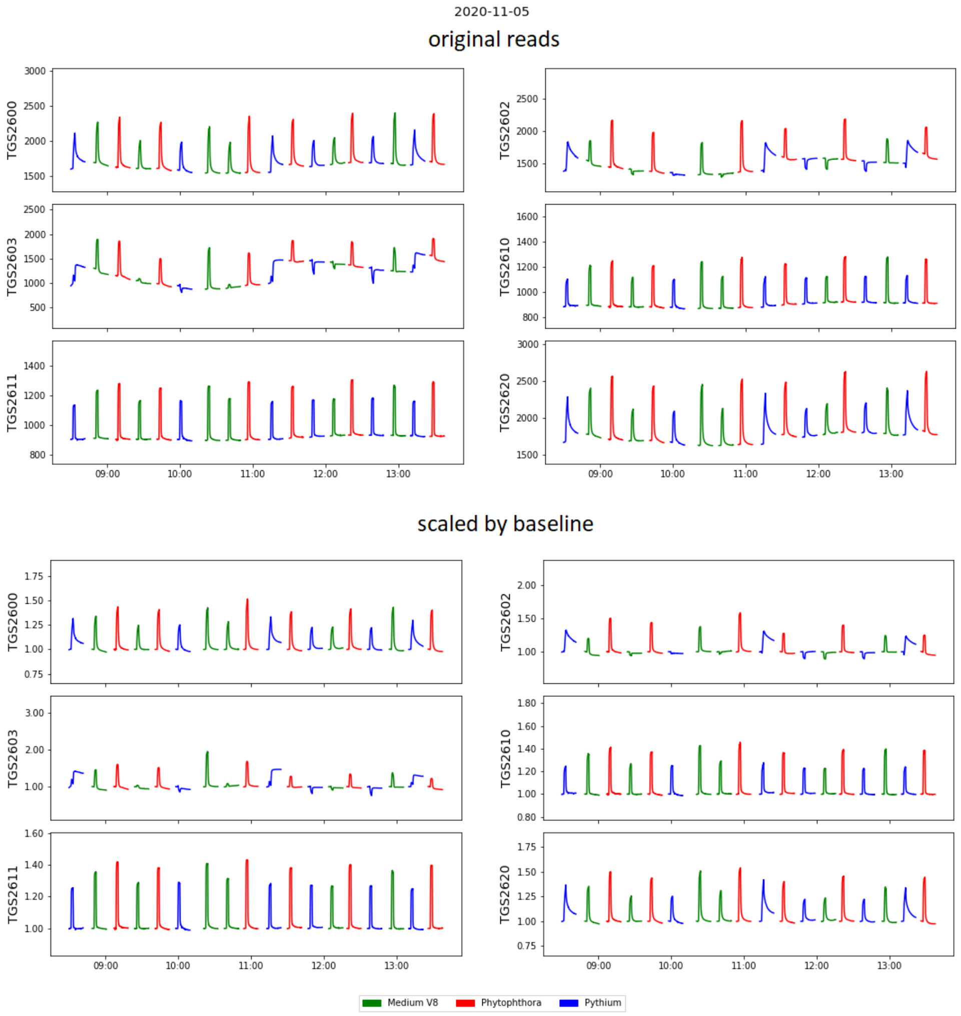

Figure S4. Sensor responses for measurements performed on 2020-11-05

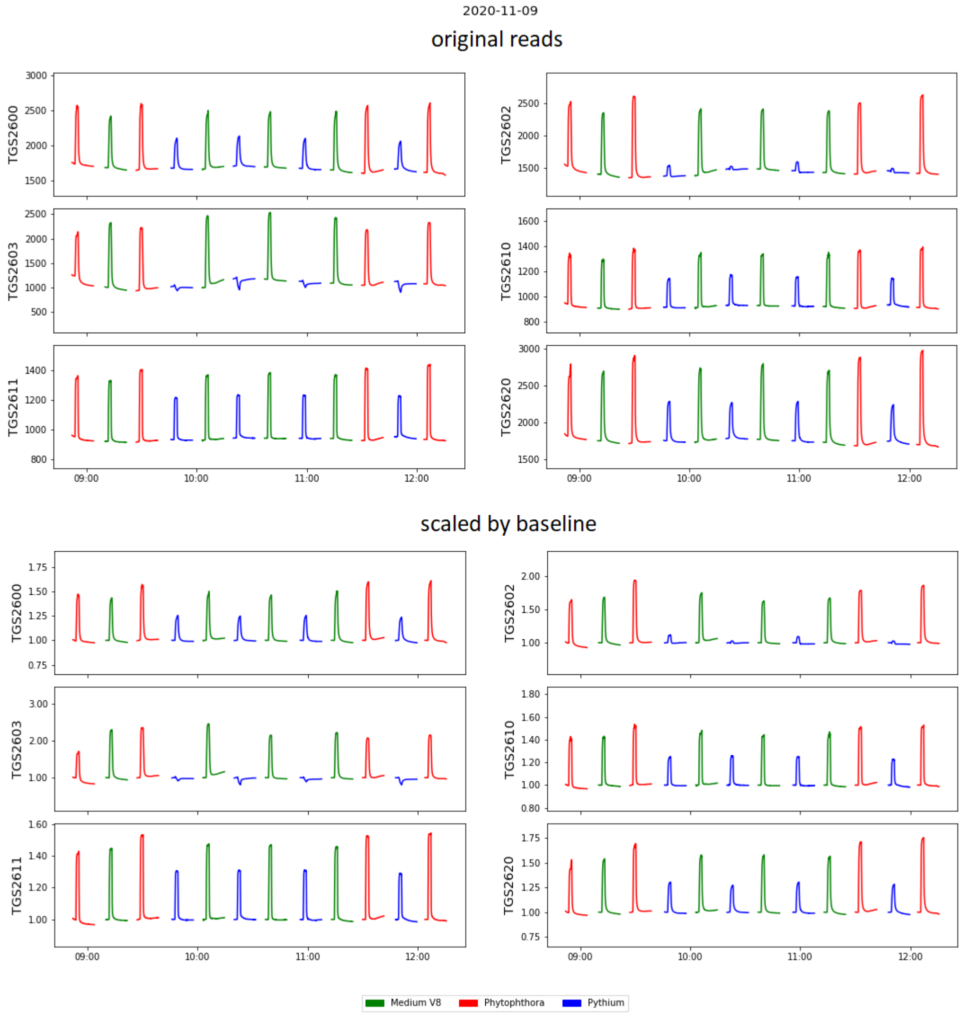

Figure S5. Sensor responses for measurements performed on 2020-11-09

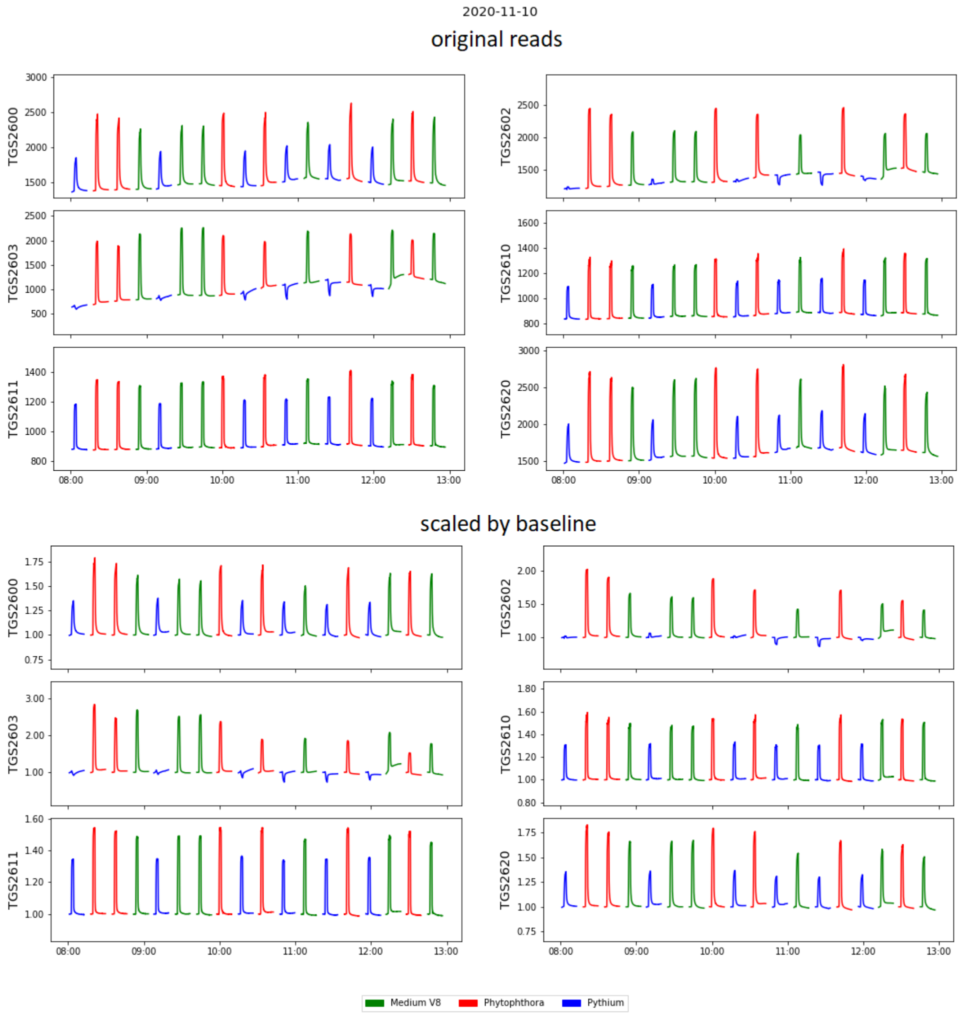

Figure S6. Sensor responses for measurements performed on 2020-11-10

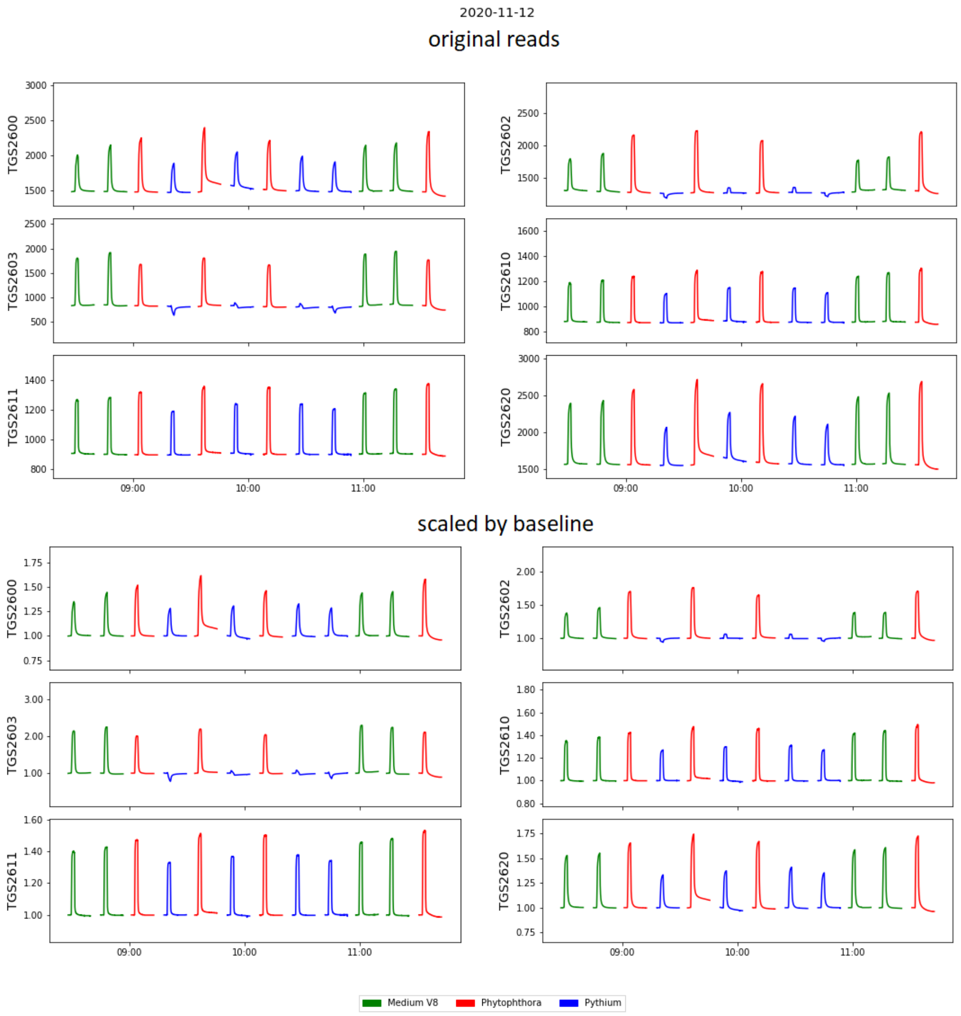

Figure S7. Sensor responses for measurements performed on 2020-11-12

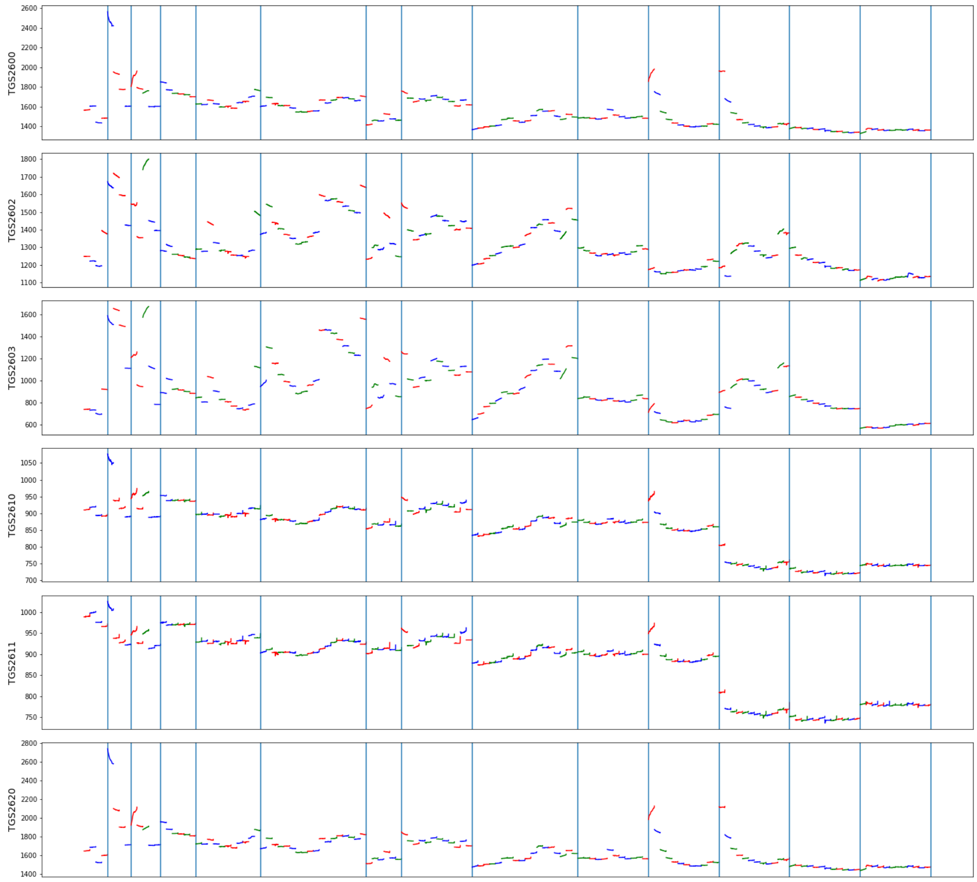

Figure S8. Response of sensors exposed to the clear air during the baseline measurement. Vertical lines separate different days of the experiment.

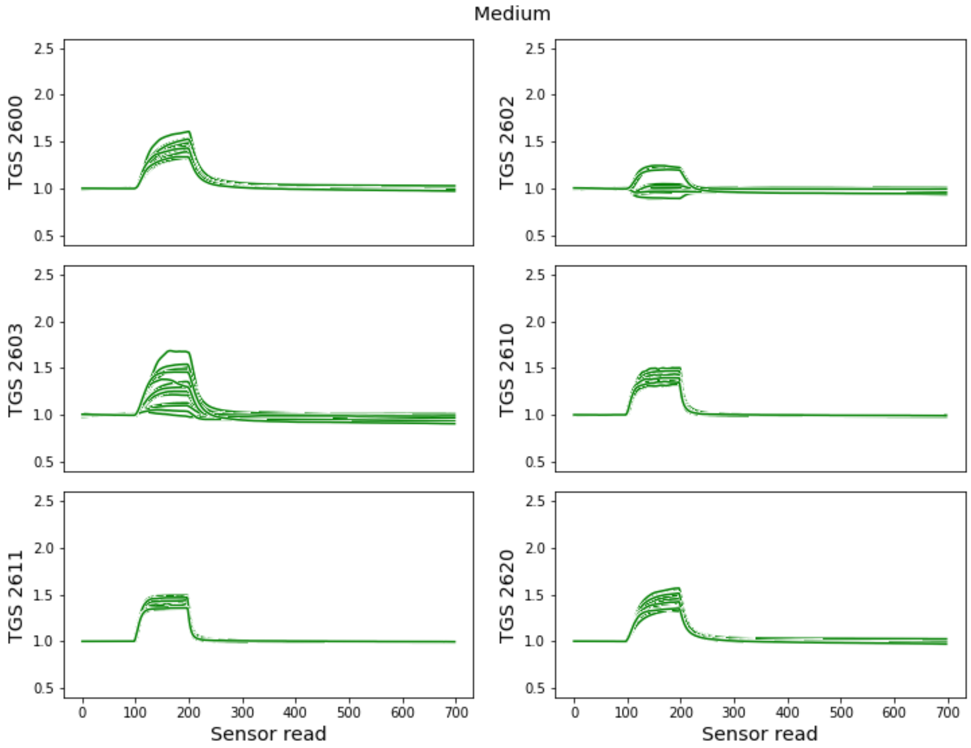

Figure S9. Several examples of sensors' responses to growth media odors for measurements on various samples during several days.

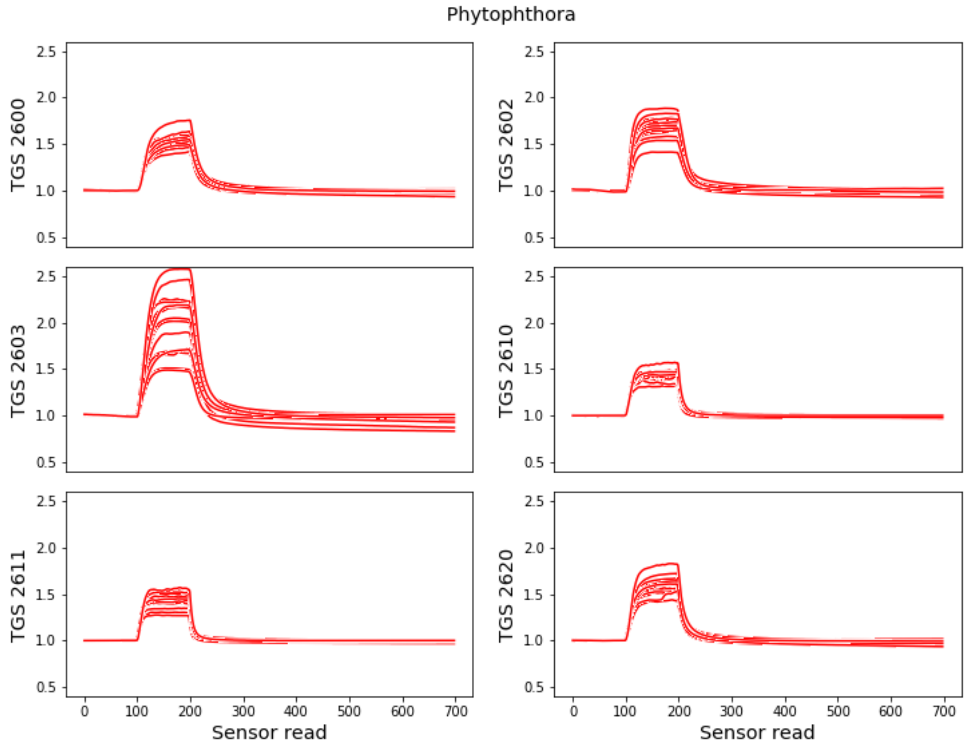

Figure S10. Several examples of of sensors' responses to *Phytophthora* odors for measurements on various samples during several days.

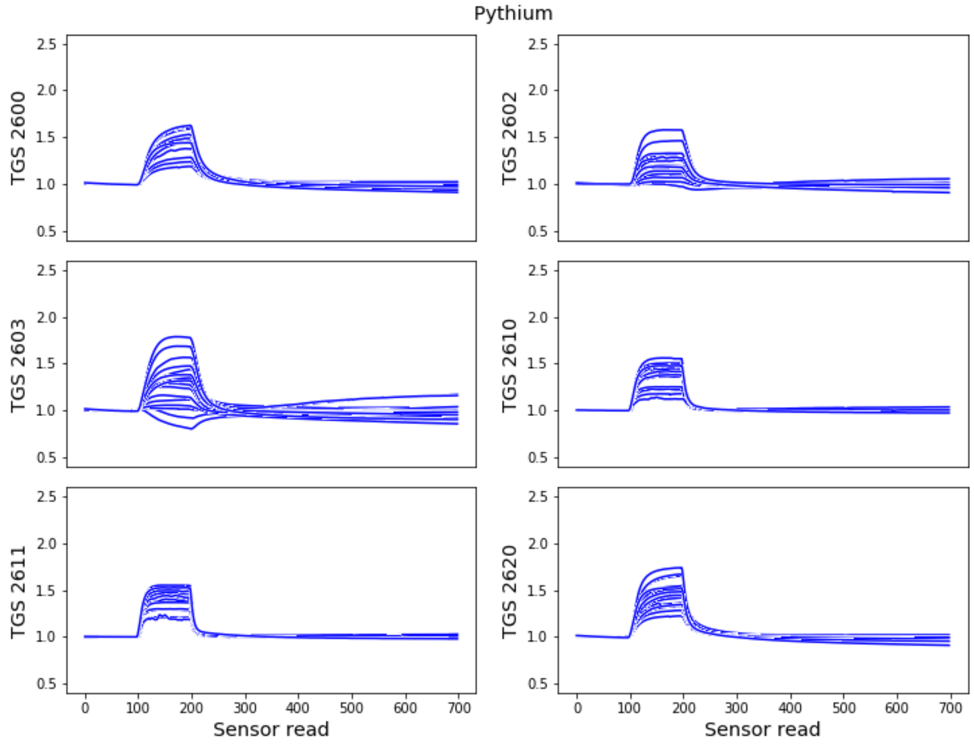

Figure S11. Several examples of of sensors' responses to *Pythium* odors for measurements on various samples during several days.
